# Kidney ventrally rotation technique in retroperitoneal robot-assisted partial nephrectomy for posterior hilar tumor: technical feasibility and preliminary results

**DOI:** 10.1186/s12957-020-01928-2

**Published:** 2020-06-30

**Authors:** Xiaotao Yin, Sinan Jiang, Zhiqiang Shao, Yongliang Lu, Jiaxiang Guo, Yi Xiao, Xiaoying Zhu, Hualiang Yu, Han Ma, Yu Yang, Jiangping Gao

**Affiliations:** 1 Department of Urology, The Forth Medical Center of the PLA General Hospital, No. 51 Fucheng Rd, Haidian District, Beijing, 100048 China; 2 Department of Urology, Linyi People’s Hospital, Linyi, China; 3 Department of Urology, First Medical Center of the Chinese PLA General Hospital, Beijing, China

**Keywords:** Robotic surgery, Partial nephrectomy, Posterior hilar tumor, Retroperitoneal approach, Kidney rotation

## Abstract

**Purpose:**

The retroperitoneal robotic assisted partial nephrectomy (RAPN) is suitable for tumors locating on the posterior side of the kidney. However, the posterior hilar tumor poses an additional surgical challenge due to the special location and poor tumor exposure. We developed a novel kidney ventrally rotation technique to overcome this difficulty during retroperitoneal RAPN and evaluated its efficacy in a retrospective case-control comparative study.

**Methods:**

From March 2016 to April 2019, a total of 39 patients with posterior renal hilar tumor underwent retroperitoneal RAPN. The kidney ventrally rotation technique, which improved the tumor exposure by opening the peritoneum and rotating the kidney ventrally, was applied in 24 cases, and the conventional RAPN was performed in the other 15 cases (control group). Perioperative data was analyzed to evaluate the efficacy of the kidney ventrally rotation technique.

**Results:**

In kidney rotation group, the 24 patients underwent RAPN successfully without converting to open surgery or radical nephrectomy. The warm ischemia time was 17.4 ± 6.6 min, which was significantly shorter than 24.5 ± 8.3 min in control group. The mean operation time (80 ± 24 min) and estimated blood loss (104 ± 65 ml) were not different from the control group. No sever complications occurred, and no positive surgical margin was found in all the malignant cases. After 14 months follow-up, no recurrence or metastasis occurred in all cases.

**Conclusion:**

Kidney ventrally rotation technique is safe and feasible for improving the exposure of posterior renal hilar tumor during retroperitoneal RAPN. It could be regarded as an efficient option for the management of posterior hilar tumor.

## Introduction

Robot-assisted laparoscopic partial nephrectomy (RAPN) is demonstrated to be superior to conventional laparoscopic partial nephrectomy (LPN) in terms of estimated blood loss and warm ischemia time, because of the 3D vision and precise dissection of the robotic system [1, 2]. Usually, the choice of operation approach during RAPN mainly depends on the tumor location, for example, transperitoneal approach for anterior tumor and retroperitoneal approach for posterior tumor [3]. The surgical management of posterior renal hilar tumor or posterior lip tumor is still a difficult challenge for urologists. The posterior hilar tumor locates behind the hilar vessels, so the retroperitoneal approach may be more direct and suitable without interference of hilar vessels. However, the exposure of posterior hilar tumor during retroperitoneal RAPN is still difficult in some cases because of the narrow retroperitoneal space, even for the experienced surgeon. Therefore, we propose a novel efficient kidney ventrally rotation technique, which improves the exposure of the posterior hilar tumor by opening the ventral peritoneum and rotating the kidney ventrally. In this article, we would describe this technique and evaluate its feasibility and efficacy in a retrospective case-control comparative study including patients who underwent retroperitoneal RAPN for posterior hilar tumor.

## Methods

### Patients

**Fig. 1:**
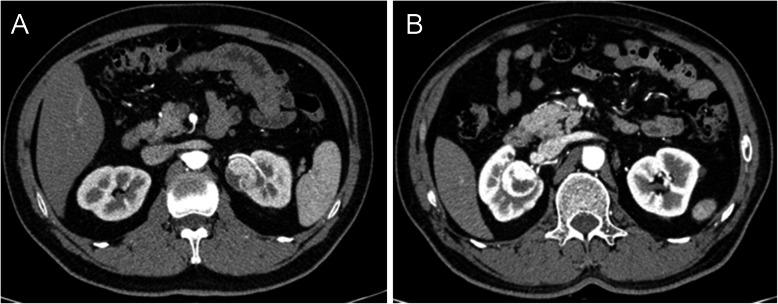
**a** Renal-enhanced computed tomography scan showing posterior hilar tumor (left side). **b** Renal-enhanced computed tomography scan showing posterior hilar tumor (right side)

From March 2016 to April 2019, the patients with posterior hilar tumor (≤ 7 cm) that underwent retroperitoneal robotic-assisted partial nephrectomy were first screened. The posterior renal hilum tumor was defined as the tumor locating in the posterior renal hilum region and being close to the main renal vessels (Fig. 1). The cases that used a third robotic arm and instrument or a second hand-assisted trocar were excluded. Finally, 24 cases that were applied kidney ventrally rotation technique were included in the kidney rotation group, and 15 cases that underwent conventional retroperitoneal RAPN were included in control group. The preoperative computed tomography (CT) or magnetic resonance imaging (MRI) examination was evaluated to obtain the tumor parameters, including tumor size, tumor location, and and tumor complexity according to R.E.N.A.L nephrometry score [4]. All cases were diagnosed as single tumor on the posterior side of the renal hilum with no lymph node or renal vessel involvement. The metastatic cases were excluded by routine chest x-ray or other specific scan according to clinical indication.

In our hospital, the patients were all informed that their clinical data might be used in future study without invasion of privacy during hospitalization. And this study was approved by the ethical committee of Forth Medical Center of the Chinese PLA General Hospital. The data of patients’ demographic characteristics, tumor sizes, R.E.N.A.L scores, preoperative laboratory results, warm ischemic time, estimated blood loss, operation related complications, and pathologic results were collected retrospectively. And all the patients were followed postoperatively according to the recommendation of the EAU guideline [5].

### Operation procedure

All operations were performed by the same surgeon with DaVinci Si surgical system. After adopting total anesthesia with tracheal intubation, the patients were positioned in the full-flank lateral decubitus position. Patients’ affected sides were upward and vertical to the operation bed. The retroperitoneal space was established by modified method of finger dissection [6]. First, the skin and subcutaneous tissue at 2 cm above the iliac crest on the mid-axillary line were incised for 2 cm. Then, the muscle and lumbodorsal fasciae were dissected with vessel forceps, and the retroperitoneal space was dissected bluntly with finger. A 12-mm trocar was placed at this site for camera. Under the guidance of the lens, two 8 mm trocars were placed about 2 cm above the plane of the camera trocar along the anterior and posterior axillary line respectively. Another 12 mm trocar was placed 2 cm above the superior spine as assistance port. The robot enters from the longitudinal direction of the patient’s head side, then the machine docking was completed.

**Fig. 2:**
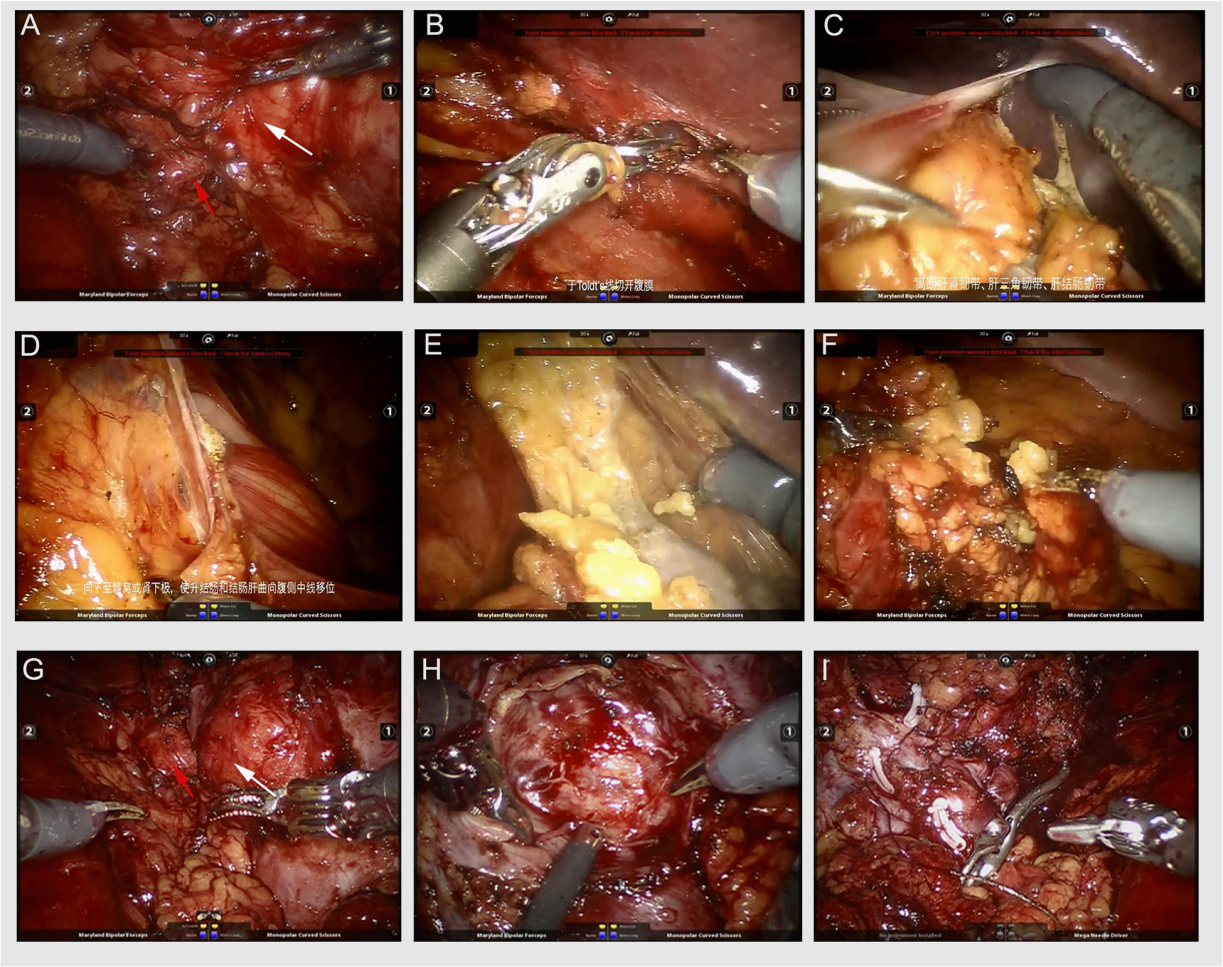
The procedures of kidney ventrally rotation technique for posterior hilar tumor. **a** The posterior hilar tumor (white arrow) was adjacent to the renal artery (red arrow) and could not be exposed thoroughly. **b** The ventral peritoneum was incised at the weakness. **c** Extend the peritoneum incision up to the upper pole of kidney and cut the triangular ligament and the hepatocolic ligament if necessary. **d** Extend the peritoneum down to the lower pole of the kidney. **e** Cut the fat tissue of the upper pole of the kidney. **f** Cut the fat tissue of the lower pole of the kidney. **g** Rotate the kidney ventrally and improve the tumor exposure, thus the anatomic relationship between tumor (white arrow) and renal artery (red arrow) was exposed clearly. **h** The tumor bed after resection or enucleation, which was convenient to be sutured. **i** The kidney after suture completion

The perirenal fasciae were incised longitudinally after the extraperitoneal fat was removed. The renal artery and vein were dissected, and the posterior hilar tumor could not be exposed with satisfaction (Fig. 2a).

The conventional retroperitoneal RAPN was performed by direct tumor resection and kidney suture, while the kidney ventrally rotation technique was performed as follows.

### Kidney ventrally rotation procedure

**Fig. 3:**
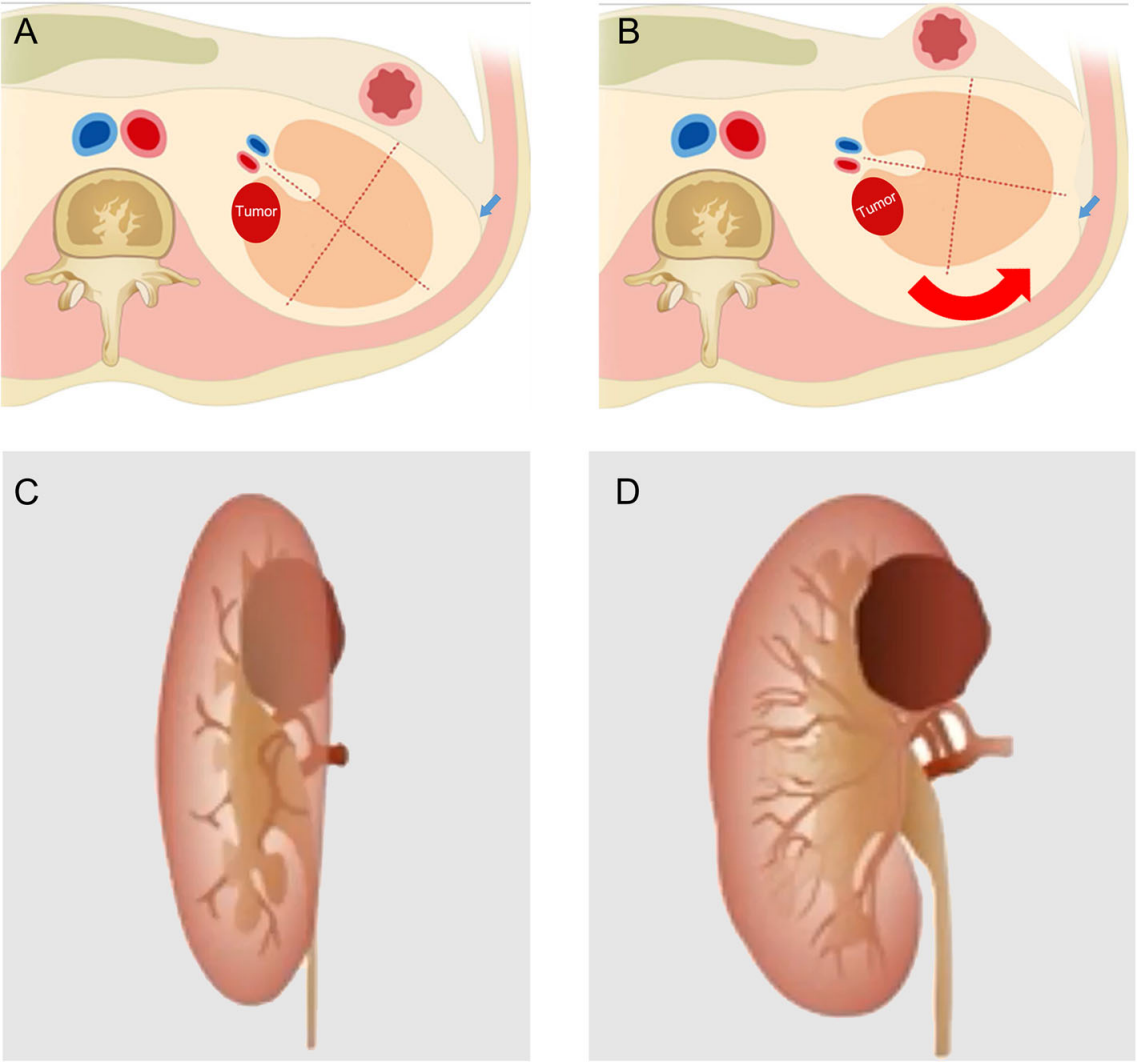
The schematic of the kidney ventrally rotation technique. **a** The tumor located at the posterior side of the renal hilum, which was not at the center of the operation field during retroperitoneal RAPN. The wrinkles or weakness of the peritoneum was selected as the incising site (blue arrow). **b** The ventral peritoneum was extended cephalad and caudally, and the kidney was rotated ventrally. Consequently, the tumor exposure was improved enough for the further resection and suture. **c** The longitudinal view of the posterior hilar tumor during retroperitoneal RAPN. **d** The improved exposure of the tumor after kidney ventrally rotation

First, incise the peritoneum at the wrinkles or weakness (Toldt’s line) (Fig. 2b), up to the hepatic flexure of colon for right kidney, even cut the triangular ligament, and the hepatocolic ligament if necessary (Fig. 2c). And for the left kidney, incise the peritoneum up to the spleen upper edge and cut the splenorenal ligament and splenocolic ligament if necessary. Then, the peritoneum was cut down to the lower edge of the kidney (Fig. 2d). The posterior side of the kidney was separated along the space between the renal parenchyma and the perirenal fat, and the adipose tissue of the upper (Fig. 2e) and lower (Fig. 2f) poles of the kidney is cut off respectively. The intraperitoneal bowel could drop down to the contralateral side due to the gravity on lateral position, and the kidney could be also ventrally rotated automatically or simply by retracting. Thus, the posterior hilar tumor could be exposed to the center of the surgical field without bowel interfering (Fig. 2g). The schematic of the kidney ventrally rotation technique was shown in Fig. 3. And the videos of kidney ventrally rotation technique were also uploaded on the website (Additional files).

**Additional file 1.** Incise the peritoneum to upper pole of the kidney.

**Additional file 2.** Incise the peritoneum to lower pole of the kidney.

**Additional file 3.** Cut the fat tissue of the upper pole of the kidney.

**Additional file 4.** Cut the fat tissue of the lower pole of the kidney.

**Additional file 5.** Rotate the kidney ventrally and block the renal vessels.

Partial nephrectomy is performed after routine clamping of the renal artery (and, if necessary, the renal vein). Stitching is performed using a knot-free barbed thread continuous method (Fig. 2h, i).

## Results

Patients and tumors characteristics were summarized in Table 1. The kidney rotation group included 18 males and 6 females, with mean age of 52.6 ± 14.5 years (range 26–78 years). The mean body mass index was 25.67 ± 3.94 kg/m^2^ (range 18.64–35.49 kg/m^2^). The mean tumor size was 4.3 ± 1.7 cm (range 2.5–8 cm). The median R.E.N.A.L score was 9 (range 7–12). All the 24 patients underwent RAPN successfully without converting to open surgery or radical nephrectomy. The control group included 10 males and 5 females, with mean age of 62.5 ± 9.4 years, mean BMI of 24.64 ± 4.21 kg/m^2^, mean tumor size of 3.8 ± 1.6 cm, and median R.E.N.A.L score of 8.

**Table 1:** Demographic and tumor characteristics

Variables	Kidney rotation	Control
Patients, *n*	24	15
Age, yr, mean (SD)	52.6 (14.5)	62.5 (9.4)
Male gender, *n* (%)	18 (75.0)	10 (66.7)
BMI, mean (SD)	25.67 (3.94)	24.64 (4.21)
RENAL score, median (range)	9 (7–12)	8 (7-11)
Tumor size, cm, mean (SD)	4.3 (1.7)	3.8 (1.6)

*BMI* body mass index, *CCI* Charlson comorbidity index, *SD* standard deviation.

The perioperative outcomes were listed in Table 2. The warm ischemia time was 17.4 ± 6.6 min in the kidney rotation group, which was significantly shorter than 24.5 ± 8.3 min in the control group (*P* < 0.05). There were no significant differences between the kidney rotation group and control group in operation time and the estimated blood loss (*P* > 0.05). In the kidney rotation group, no abdominal organ damage, vascular injury, or subsequent bleeding occurred. Pathological results showed 18 cases of clear cell carcinoma, 4 cases of angiomyolipoma, 1 case of papillary carcinoma, and 1 case of oncocytoma. No positive surgical margin was found in all cases. After the operation, all the patients were followed as the recommended schedule, and the median follow-up time was 14 months. No recurrence or metastasis occurred in all patients.

**Table 2:** Perioperative outcomes

Variables	Kidney rotation (*n* = 24)	Control (*n* = 15)	*P*
WIT, min (SD)	17.4 (6.6)	24.5 (8.3)	*
EBL, ml (SD)	104 (65)	86 (60)	-
Operation time, min (SD)	80 (24)	83 (30)	-
Operation conversion, *n* (%)	0 (0%)	0 (0%)	-
Positive surgical margin, *n* (%)	0 (0%)	0 (0%)	-
Median follow-up, mon (median)	14	18	-
Recurrence or metastasis, *n* (%)	0 (0%)	0 (0%)	-
Final pathology			-
Clear cell carcinoma, *n* (%)	18 (75.0)	12 (80.0)	
Papillary carcinoma, *n* (%)	1 (4.2)	1 (6.7)	
Oncocytoma, *n* (%)	1 (4.2)	0 (0)	
Angiomyolipoma, *n* (%)	4 (16.7)	2 (13.3)	

*WIT* warm ischemia time, *EBL* estimated blood loss, *SD* standard deviation

*P < 0.05, -*P* > 0.05

## Discussion

Multiple studies have demonstrated a comparable cancer-specific survival for PN vs RN-treating pT1 RCC [7, 8]. In addition, PN demonstrated better preserved kidney function, thereby potentially lowering the risk of development of cardiovascular disorders [9, 10]. So, PN is increasingly becoming a preferred choice for surgeon and patients with confined renal tumor. Robotic surgical system could shorten the learning curve and warm ischemia time, because of its unique three-dimensional vision, precise operation, and flexible instruments. As for oncological outcomes, many studies have indicated that the RAPN has no significant differences with LPN or open PN, while RAPN is superior to LN and OPN in terms of estimated blood loss, warm ischemia time, hospital stay, and preserved effective nephron [11].

Like conventional LPN, RAPN usually has two conventional approaches: transperitoneal and retroperitoneal, depending on the location of the tumor [3]. Chinese urologists are more familiar with the retroperitoneal anatomy, as retroperitoneal LPN is predominant in most Chinese hospitals. Especially for tumors located on the posterior side of kidney, retroperitoneal approach is more suitable because of the direct access to tumor without the excess disturbance of the abdominal organs. And some studies have demonstrated that retroperitoneal approach has the advantages of patients’ quicker recovery from operations and less postoperative complications.

Even with the application of robots, the surgical difficulty of renal hilar tumors is still significantly higher than that of non-hilar tumors [12, 13]. The average operation time and renal ischemic time is longer, intraoperative blood loss is more, and the rate of intraoperative conversion to radical resection is higher for hilum tumor [14]. Posterior hilar or lip tumor poses additional technical challenges to the operating surgeon. Conventional transperitoneal approach may not be suitable despite of the large operation space, because the tumor is located behind hilar vessels, which interferes with the whole tumor dissection and renorrhaphy process. Retroperitoneal approach is more direct and appropriate for posterior hilar tumor, but the tumor exposure may be still unsatisfied in some cases which would hinder the tumor resection and suture processes. We also attempted to rotate the kidney ventrally without incising the peritoneum after dissociating the kidney completely from the fat layer during RARN, but the degree of rotation was limited and the improvement of tumor exposure was not satisfied. Some studies used the fourth mechanical arm on the ventral side for renal traction fixation during retroperitoneal RAPN, which could reduce the complications and margins caused by poor exposure and unclear vision [15]. However, this method consumed extra instrument or assistant, which increased the operation cost.

In this study, we freed the kidney by opening the peritoneum and rotated the kidney ventrally, which could maximally expose posterior hilar tumors. Among our 24 cases with posterior hilar tumor, the mean tumor size was 4.3 ± 1.7 cm, and the median R.E.N.A.L score was 9, which indicated the difficulty and complexity in these operations. Compared with the conventional method, the kidney ventrally rotation technique significantly improved the tumor exposure and reduced the difficulty of tumor resection and suture process, which achieved a shorter warm ischemia time (17.4 min vs 24.5 min, *P* < 0.05). Besides, the kidney ventrally rotation process was not complicated or time-consuming. The whole operation time in the two groups was not significantly different (*P* > .05). In the kidney rotation group, no case was converted to radical nephrectomy or open surgery, and no positive surgical margin or other postoperative major complications occurred. Moreover, this technique could also be adopted in conventional retroperitoneal LPN. In summary, although this technique attenuated the isolation character of retroperitoneal space, it facilitated the management of the posterior hilar tumor during retroperitoneal RAPN significantly, especially for surgeons without extensive surgical experience. We summarized four important steps which should be emphasized.

### Incising peritoneum

The incision location of the peritoneum was selected in the weak or wrinkled place, such as the Toldt’s line. Then, the incision must be extended cephalad and caudally along the paracolic sulcus under the direct vision. The incision extension could be adjusted during the whole operation depending on the tumor exposure. It was necessary to observe the abdominal organs during the incision process to avoid accidentally injuring, such as the intestine, liver, spleen, pancreas, and the diaphragm. The patient with history of abdominal surgery or intensive abdominal adhesion may not be suitable for this technique, because they had the increased risk of organ injury.

### Rotating the kidney

As the kidney is fixed by the surrounding adipose tissue, simply cutting the peritoneum would not free the kidney adequately. Therefore, it is necessary to free the kidney by dissecting adipose tissue of upper and lower poles of the kidney, so that the kidney can be automatically rotated ventrally or simply by kidney retraction. As followed, the posterior hilar tumor would be turned towards surgical filed and fully exposed, which could decrease the surgical difficulty significantly.

### Resection

The tumor resection is another technical challenge in partial nephrectomy for posterior hilar tumor. By kidney ventrally rotation method, the tumor exposure could be improved, which is the prerequisite for successful tumor resection. Because the tumor is close to renal vessels and collecting system, the accidental damage should be avoided during the resection process. The tumor enucleation technique could be used, which dissects the tumor mainly by blunt excision along tumor pseudocapsule. This technique does not increase the risk of positive margin, which has been reported in some other studies [16]. If necessary, 3D reconstruction using enhanced CT or MRI scan data could also be applied to understand the tumor location, depth, and the relationship with blood vessels or collecting system.

### Renorrhaphy

For the hilar tumor, the suture method for conventional tumor may be not suitable. The parenchyma edge of tumor bed after resection is close to renal sinus, which contains main branches of renal vessels and collecting system. Besides, the parenchyma edge close to renal sinus is not enough thick and may be cut through by the thread when performing the conventional kidney renorrhaphy. Therefore, we recommend the ring or c-shaped suture technique in the renorrhaphy of hilar tumor, which could reduce the difficulty of renorrhaphy and maximally preserve the effective nephron [17]. It is best to start stitching from the side close to renal sinus, which ensures that the blood vessel is not damaged as much as possible.

Several limits exist in this study, including small sample size and retrospective nature, while our study proposed a novel surgical technique for posterior hilar tumors and verified its feasibility, safety, and outcome in a preliminary group of cases. And prospective and controlled study with lager sample size is needed further.

## Conclusion

Our study reports the preliminary results of kidney ventrally rotation technique in retroperitoneoscopic RAPN for posterior hilar tumor. The technique could effectively improve the exposure of posterior renal hilar tumor, consequently decreasing the difficulty and risk of the operation for tumors with this special location. The technique could be a useful and reasonable option for the management of posterior hilar tumor during retroperitoneal RAPN.

## Data Availability

The data that support the findings of this study are available from the corresponding author upon reasonable request.

## Abbreviations

PN: Partial nephrectomy
LPN: Laparascopic nephrectomy
RAPN: Robotic-assisted partial nephrectomy
OPN: Open partial nephrectomy
CT: Computerized tomography
MRI: Magnetic resonance imaging
CCI: Charlson comorbidity index
BMI: Body mass index
EBL: Estimated blood loss
WIT: Warm ishemia time
SD: Standard deviation
PSM: Positive surgical margin
RCC: Renal cell carcinoma
